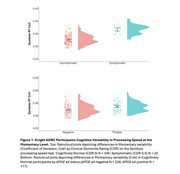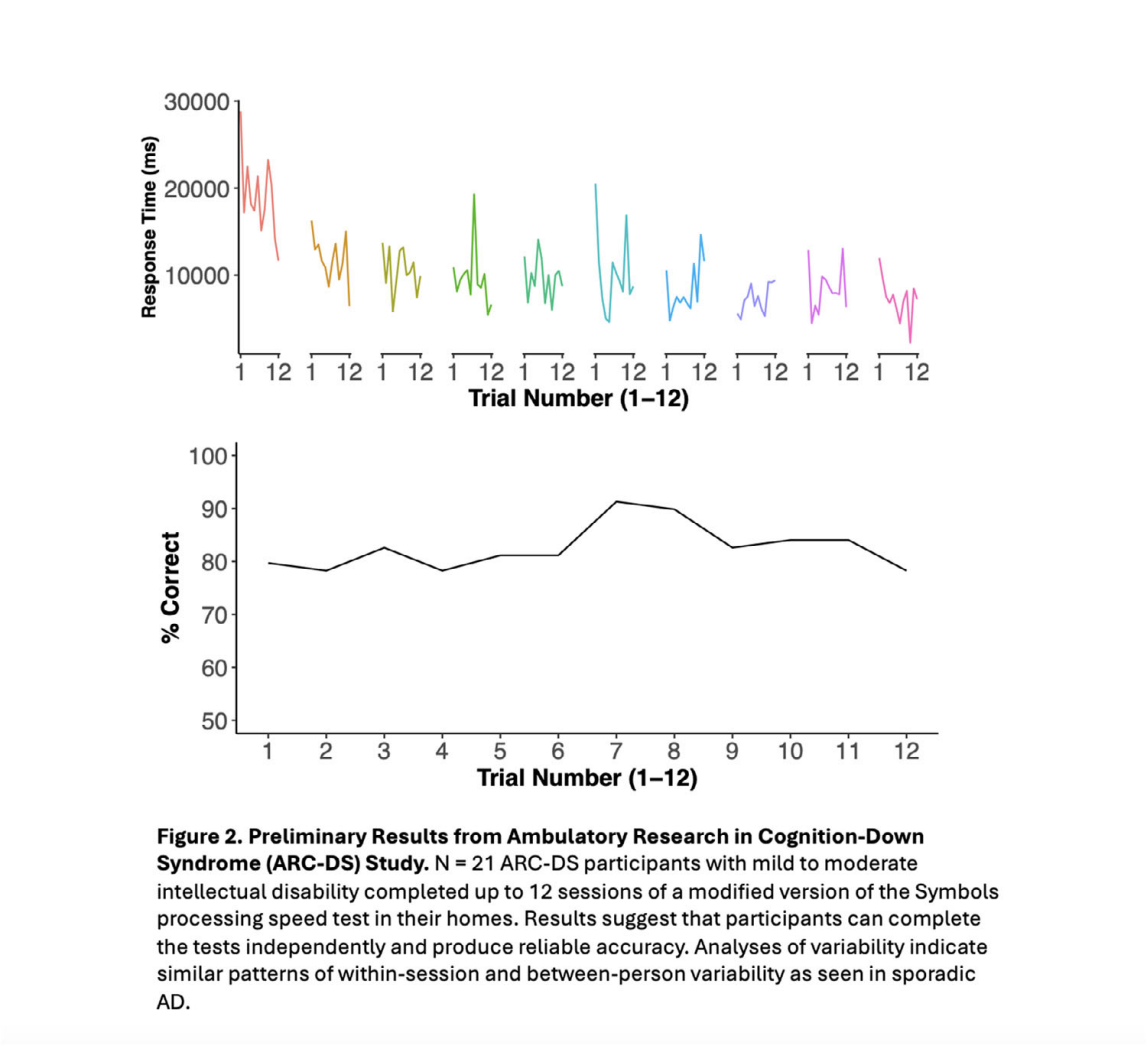# Pushing Boundaries with Remote Digital Cognitive Assessments in Alzheimer's Disease

**DOI:** 10.1002/alz70857_102259

**Published:** 2025-12-25

**Authors:** Jason J. Hassenstab

**Affiliations:** ^1^ Washington University in St. Louis, St. Louis, MO, USA

## Abstract

**Background:**

Remote technologies for cognitive assessments have evolved to such a degree that they are more than digital substitutes for conventional tests and can reveal unique aspects of cognitive changes in Alzheimer's disease (AD). Concerns about technology familiarity and usability in older adults and in populations with intellectual disability like Down syndrome‐associated AD (DSAD) are being confronted by studies showing that these populations use technology successfully, and that they are often ideal for increasing accessibility and less burdensome for participants. Here we highlight a novel cognitive outcome in sporadic AD that is only feasible with remote digital cognitive assessments and describe our emerging efforts to translate these methods to assess cognition remotely in global studies of DSAD.

**Method:**

Participants were from the Knight Alzheimer's Disease Research Center (Knight ADRC, *N* = 377) and from the Ambulatory Research in Cognition‐Down Syndrome (ARC‐DS) study (*N* = 21). Knight ADRC participants completed four ultra‐brief cognitive assessments per day for seven consecutive days in their natural environments. In the ARC‐DS preliminary study, participants with mild to moderate intellectual disability completed a modified version of the remote test up to 12 times onsite and in their homes. We analyzed variability at two different timescales: at the daily level (Did cognition fluctuate from day‐to‐day?) and at the momentary level (Did cognition fluctuate from moment‐to‐moment?).

**Results:**

In Knight ADRC participants, there were significant differences in cognitive variability across the week of testing (Figure 1a). Participants with the earliest symptoms of impairment (Clinical Dementia Rating (CDR) 0.5) demonstrated more variability at both the daily and momentary timescales than those who were cognitively normal (CN, CDR 0). In CN participants at genetic risk for AD, both daily and momentary variability were significantly greater in *APOE e4* carriers compared to noncarriers (Figure 1b). On the simplified version of the processing speed test designed for older adults with DSAD, we found that participants could complete testing independently and demonstrated similar patterns of variability across testing sessions (Figure 2).

**Conclusion:**

Remote cognitive assessments can capture unique features of cognitive change in AD and are suitable for use in all AD populations, including sporadic AD and DSAD.